# The Association between *ABCB1* C1236T/C3435T SNPs and *H. pylori* Infection among Jordanians

**DOI:** 10.3390/genes11010063

**Published:** 2020-01-05

**Authors:** Mohammed N. BaniHani, Omar F. Khabour, Karem H. Alzoubi, Nabil A. Bashir, Muhamad Ali K. Shakhatreh, Salsabeel H. Sabi, Nasr Alrabadi

**Affiliations:** 1 Department of General Surgery and Urology, Jordan University of Science and Technology, Irbid 22110, Jordan; 2 Department of Medical Laboratory Sciences, Jordan University of Science and Technology, Irbid 22110, Jordan; Khabour@just.edu.jo (O.F.K.); mkshakhatreh@just.edu.jo (M.A.K.S.); asharmohammad63@yahoo.com (S.H.S.); 3 Department of Clinical Pharmacy, Jordan University of Science and Technology, Irbid 22110, Jordan; 4 Department of Physiology and Biochemistry, Jordan University of Science and Technology, Irbid 22110, Jordan; nbashir@just.edu.jo; 5 Department of Pharmacology, Faculty of Medicine, Jordan University of Science and Technology, Irbid 22110, Jordan; nnalrabadi@just.edu.jo

**Keywords:** *H. pylori*, MDR1, *ABCB1*, Jordan, gastric, SNPs

## Abstract

Infection with *Helicobacter pylori* (*H. pylori*) is very common and affecting about 50% of the worldwide population. Several genetic variations have been implicated in determining the clinical susceptibility to this infection. In the current study, we examined the association between C1236T (rs1045642) and C3435T (rs1045642) single nucleotide polymorphisms (SNPs) in the *ABCB1* gene and the prevalence of *H. pylori* infection among Jordanians. A total of 412 subjects (257 *H. pylori*-positive cases and 155 *H. pylori*-negative controls) were recruited and participated in the study, and the genotyping of the *ABCB1* gene was performed using RFLP-PCR techniques. A significant association was detected between C1236T and *H. pylori* infection (*p* < 0.01). The frequency of CT genotype was significantly higher in the positive cases (40.1%) compared to the controls (21.3%). In addition, the C3435T SNP was weakly associated with *H. pylori* infection (*p* = 0.077). Haplotype analysis of C1236T and C3435T SNPs showed that the TT haplotype was present in 22.7% of the positive cases compared to 30.7% of the negative controls (*p* < 0.05, odds ratio = 0.663, 95% CI: (0.483–0.911)). Consequently, the TT haplotype seems to decrease the risk of *H. pylori* infection. In conclusion, the current results suggest an association between *ABCB1* SNPs and *H. pylori* infection in the Jordanian population.

## 1. Introduction

*H. pylori* infection is one of the most common bacterial infections, affecting approximately half of the world population [1,2]. It can cause chronic gastritis, which in turn may be complicated with peptic ulcer disease, lymphoma, and gastric adenocarcinoma [3,4]. The major factor that affects the rates of cure from *H. pylori* infection is the sufficient inhibition of acid secretion using the anti-secretory agents such as proton pump inhibitors (PPIs) [5,6]. However, the incidence of *H. pylori* infection and its sufficient eradication using pharmacological therapies are also affected by genetic factors, such as those affecting inflammatory cytokines and variants of the genes encoding drug-metabolizing enzymes and drug transporters [1,7,8].

The multi-drug resistance gene (*ABCB1*) is a gene that maps to chromosome 7 and encodes for P-glycoprotein (P-gp), which is a large transmembrane protein of 170 Kd and functions as an ATP-dependent efflux pump [9]. The gene is also highly expressed in normal tissues such as brain, liver, kidney, lymphocytes, placenta, gut, and testes [10]. P-gp plays an important role in regulating absorption, distribution, and the elimination of drugs [11]. *ABCB1* has also been found to mediate the energy-dependent efflux of xenobiotics in epithelial tissues throughout the human body [12]. On the other hand, the expression of the *ABCB1* gene is highly variable among subjects of the same and different races, and many variants have been identified in its genetic sequence [9].

Several studies have investigated the relationship between *ABCB1* polymorphisms and *H. pylori* infection [13,14,15]. For instance, two Australian studies showed a strong association between them [16,17]. In other studies from Japan, the methylation status of the *ABCB1* gene was found to be strongly associated with *H. pylori* infection and gastric cancer [18,19]. In addition, *ABCB1* polymorphisms were found to be associated with gastric cancer and were identified as one of the determinants for the successful eradication of *H. pylori* using triple therapy with lansoprazole, amoxicillin, and clarithromycin [20]. Similar findings were reported in a study that was conducted on Polish patients [21]. However, reports on the association between *ABCB1* polymorphisms and *H. pylori* infection from Arabic populations are still lacking. Therefore, in the current investigation, the association between *ABCB1* polymorphisms (C1236T: rs1045642 and C3435T: rs1045642) and *H. pylori* infection was examined.

## 2. Materials and Methods

The study was first approved by the Institutional Review Board of Jordan University of Science and Technology (approval number: 16/6/4/3141, date: 18-10-2017). Patients (>18 years old) who were scheduled to undergo a gastroscopic examination at King Abdullah University Hospital (KAUH) were invited to participate in the study. Recruitments were from September 2017 to July 2019. All participants gave informed consent after a full explanation of the study objectives and procedures. Patients diagnosed with non-ulcer dyspepsia as established during gastroscopy with normal findings or mild gastritis were included in the study. On the other hand, patients with a history of gastric surgery or those who were taking medications in the preceding four weeks (such as anticoagulants, antibiotics, and proton pump inhibitors) were excluded from the study [22,23]. Participants’ demographics such as age, gender, height, and weight were collected using a structured questionnaire, and their clinical history was obtained from their medical files and records.

### 2.1. Diagnosis of *H. pylori* Infection

The diagnosis of *H. pylori* infection was performed using biopsy samples from gastric antrum. Biopsy sampling is a routine part of the standard diagnostic procedure for those patients who undergo gastroscopic examination at KAUH. Those samples from the gastric antrum were subjected to further laboratory testing (see below) for the diagnosis of *H. pylori* infection [24]. Those patients with positive tests were considered the positive cases, and those with negative tests were considered the negative controls.

### 2.2. Isolation of Genomic DNA

Blood samples for genomic DNA isolation were obtained in EDTA tubes. The DNA was extracted using a Promega blood kit (Madison, WI, USA) according to the manufacturer’s instructions [25,26]. Concentration of the extracted DNA was measured using a Bio-Rad SmartSpect_3000 device (Hertfordshire, UK), and the isolated DNA was stored at room temperature (20 °C) until used.

### 2.3. Genotyping of *ABCB1* Polymorphisms

Two common polymorphisms in the *ABCB1* gene—C1236T and C3435T—were genotyped. The polymerase chain reaction–restriction fragment length polymorphism (PCR–RFLP) assay was used for genotyping. PCR conditions, primer sequences, and restriction assays were conducted similar to what has been described in our previous work [27]. Restricted fragments were visualized using 2% agarose gel for both polymorphisms. All gels were documented using a Bio-Rad gel documentation system (Gel Doc 2000, Hercules, CA, USA).

### 2.4. Statistical Analysis

Data were analyzed using the SPSS package version for windows. Data were expressed as continuous variables (mean ± standard deviation) and discrete variables (counts and frequencies), and were compared using the chi square test. Association between examined SNPs and *H. pylori* infection was calculated using the SNPStats website (https://www.snpstats.net/start.htm). Three-locus haplotype frequencies were calculated using the SHEsis program. Power analysis was carried out using G*Power version 3.0.10 (Franz Faul, Universität Kiel, Kiel, Germany). Genotype distributions were analyzed for Hardy–Weinberg equilibrium. A *p*-value <0.05 was considered significant.

## 3. Results

A total of 412 subjects who agreed to participate in the study and who were referred for a gastroscopic examination as part of the diagnosis for *H. pylori* infection were included. After complete diagnosis of the patients using a rapid urease test and a histological examination, 257 subjects were confirmed to be *H. pylori*-positive (cases), whereas 155 subjects were confirmed to be *H. pylori*-negative (controls). Table 1 shows the demographics and clinical characteristics for those participants. The mean age of controls and cases were 42.6 ± 14.7 years and 40.8 ± 15.2 years, respectively (*p* > 0.05). Female subjects represent 48.7% of controls and 51% of cases. Body mass indices (BMIs) were not different between the two groups. In addition, no differences with respect to the frequencies of hypertension, diabetes, cardiovascular disease, thyroid dysfunction, and kidney/liver diseases were observed (Table 1).

The association between the SNPs of the *ABCB1* gene (C1236T and C3435T) and *H. pylori* infection is shown in Table 2. A significant association between C1236T and *H. pylori* infection was detected (*p* < 0.01). The frequency of the CT genotype was significantly higher in cases (40.1%) compared to controls (21.3%). Thus, the heterozygotes state was associated with a higher risk for *H. pylori* infection compared to the other genotypes.

With respect to C3435T SNP, a weak association was detected between this SNP and *H. pylori* infection. The TT genotype was lower, whereas TT was higher among *H. pylori*-positive patients when compared to *H. pylori*-negative ones (*p* = 0.07). When alleles were considered, the frequency of the C allele was significantly higher among *H. pylori*-positive subjects (*p* < 0.05).

Haplotype analysis of C1236T and C3435T SNPs is shown in Table 3. Among all examined haplotypes, the TT haplotype was present in 22.7% of cases compared to 30.7% of controls (*p* < 0.05, odds ratio = 0.663, 95% CI: (0.483–0.911)). Thus, the TT haplotype seems to decrease the risk of *H. pylori* infection. No significant association between the rest of the haplotypes and *H. pylori* infection was observed (*p* > 0.05, Table 3).

## 4. Discussion

In the current study, the association between *H. pylori* infection and the *ABCB1* gene polymorphisms were examined. The results showed a strong association between C1236T, G2677T/A, and *H. pylori* infection. In addition, a weak effect for C3435T on *H. pylori* infection was also observed.

*ABCB1* encodes for P-glycoprotein (P-gp), which functions as an ATP-dependent efflux pump and plays a role in the absorption, distribution, and elimination of drugs [28]. In addition, *ABCB1* has been found to mediate the efflux of xenobiotics in epithelial tissues throughout the human body including the gut [10]. However, the level of expression of *ABCB1* varies between subjects depending on certain polymorphisms that are present in the gene [9].

The results of the current study showed an association between C1236T and C3435T SNPs and *H. pylori* infection. This finding is in agreement with previous literature that examined the relationship between the *ABCB1* gene and *H. pylori* infection and the response to eradication therapies. For example, a positive relationship between C3435T SNP and *H. pylori* infection was detected among Australians, with the TT genotype being associated with a significantly lower P-gp expression compared with the CC genotype [16]. In addition, differential modulation of P-gp expression was observed in the TT genotype carriers between *H. pylori*-negative and *H. pylori*-positive patients [16]. Thus, a low expression of P-gp in the stomach epithelium might be associated with an increased risk of *H. pylori* infection. In agreement with those findings, the methylation status of the *ABCB1* gene was found to be strongly associated with *H. pylori* infection [18,19]. Furthermore, the C3435T polymorphism of MDR1 has been shown to influence *H. pylori*-related inflammatory conditions in the stomach [17] and to increase the risk of gastric cancer among Iranian and Japanese subjects [20,29,30]. Finally, the C3435T SNP has been shown to be associated with clinical outcomes in gastric cancer patients treated with postoperative adjuvant chemotherapy [31] and *H. pylori* eradication therapy [32]. Therefore, it can be postulated that the C1236T and G2677T/A SNPs could be influencing the course of *H. pylori* infection via modulating the expression of P-gp and/or the methylation status of corresponding genes leading to changes in inflammatory processes in the stomach.

With respect to C1236T SNP, the current report is pioneering in that the association of these variants with *H. pylori* infection is defined. However, the clinical significance of C1236T in gastric diseases is not yet well studied. In a previous study, *ABCB1* C1236T has been shown to be correlated with gastric cancer progression [33]. Thus, more studies are needed to confirm the present findings.

Other SNPs in the *ABCB1* gene has been shown to be associated with gastric diseases. For example, a study that was conducted in China showed that G2677T/A SNP was a beneficial predictor of clinical treatment outcomes in gastric cancer [34]. Similarly, a G2677T/A SNP was shown to be associated with the chemosensitivity of paclitaxel in gastric cancer [35]. The G159T, A3073C, and A1564T SNPs were found to be associated with gastric cancer risk [36,37]. Thus, *ABCB1* genetic variations seem to play a role in the susceptibility to *H. pylori* infection and the subsequent development of gastric cancer.

The findings that *ABCB1* SNPs are associated with *H. pylori* infection are important, as this infection is found in approximately 60% of the world population [38]. Globally, gastric cancer is still one of the leading causes of cancer-related deaths worldwide [39,40]. In most of the cases, gastric cancer is often asymptomatic in its early stages and by the time symptoms starts, the cancer has often reached an advanced stage and metastasized to other parts of the body [41]. Understanding the risk factors of *H. pylori* infection will help in the management of the disease and the subsequent prevention of gastric cancer.

In the current study, we attempted also to examine the role of the *ABCB1 SNPs* in the eradication of *H. pylori* using the triple therapy regimen. However, most of the recruited patients (>90%) showed complete eradications, so such analysis was not feasible. Increasing the sample size in future studies might allow for such an analysis. In addition, we did not examine the relationships between SNPs and the pathogenesis of chronic stomach issues or the methylation status of *ABCB1* DNA or how such methylation might affect the expression of the *ABCB1* gene. Moreover, for diagnosis of *H. pylori* infection, we sampled antrum only and used it, in addition to immunoglobulin testing, as a base for *H. pylori* diagnosis. Previous studies have shown that, in some cases, *H. pylori* may migrate towards the corpus [42]. Therefore, the presence of some false negatives among *H. pylori*-negative patients cannot be excluded. Overcoming such limitations in future studies is strongly recommended.

In conclusion, *ABCB1* SNPs seem to be associated with *H. pylori* infection in the Jordanian population. A strong association between C1236T and *H. pylori* infection was detected, whereas C3435T SNP showed a weak effect.

## Figures and Tables

**Table 1:** Demographics and clinical characteristics of participants.

Variable	Controls	Cases	*p* Value
Age	42.6 ± 14.7	40.8 ± 15.2	0.239
Gender			
male	64 (41.3)	126 (49.0)	
Female	91 (48.7)	131 (51.0)	0.153
BMI	28.1 ± 4.5	28.5 ± 5.2	0.503
Hypertension	25 (16.1)	57 (22.2)	0.161
Diabetes	14 (9.0%)	35 (13.6)	0.209
Cardiovascular Diseases	4 (2.6)	10 (3.9)	0.582
Thyroid Disease	87 (5.1)	8 (3.5)	0.588
Kidney Diseases	2 (1.3)	3 (1.2)	0.912
Liver Diseases	1 (0.6)	5 (1.9)	0.286

**Table 2:** Association between SNPs and *H. pylori* infection as calculated using SNPStats.

MDR1 SNP	Cases	Controls	Odds Ratio (95% CI)	*p*-Value
C1236T				
CC	81 (31.5%)	56 (36.1%)	1.00	
CT	103 (40.1%)	33 (21.3%)	0.35 (0.21–0.59)	
TT	73 (28.4%)	66 (42.6%)	0.76 (0.47–1.23)	<0.001
Allele C	265 (52%)	145 (47%)		
Allele T	249 (48%)	165 (53%)	1.21 (0.91–1.61)	<0.001
C3435T				
CC	70 (27.3%)	59 (38.1%)	1.00	
TC	134 (52.1%)	70 (45.2%)	0.62 (0.39–0.97)	
TT	53 (20.6%)	26 (16.8%)	0.59 (0.33–1.06)	0.070
C	274 (53.3%)	188 (0.61%)		
T	240 (46.7%)	122 (39%)	0.74 (0.55–0.98)	0.039

**Table 3:** Haplotype analysis.

Haplotype C1236T/C3435T	Case (Frequency)	Control (Frequency)	Pearson’s *p*	Odds Ratio (95%CI)
CC	157.24 (0.306)	92.79 (0.299)	0.84	1.032 (0.759–1.403)
TC	107.76 (0.210)	52.21 (0.168)	0.14	1.310 (0.909–1.888)
TT	116.76 (0.227)	95.21 (0.307)	0.01	0.663 (0.483–0.911)
CT	132.24 (0.257)	69.79 (0.225)	0.29	1.192 (0.856–1.662)
